# What Is the Current State of Stem Cell Therapy in Diabetes?

**DOI:** 10.3390/cells15100907

**Published:** 2026-05-15

**Authors:** Estera Bakinowska, Wojciech Jerzy Biniek, Kajetan Kiełbowski, Kamil Dyrka, Konrad Szewczyk, Hanna Ostałowska, Zuzanna Leciej, Andrzej Pawlik

**Affiliations:** 1 Department of Physiology, Pomeranian Medical University, 70-111 Szczecin, Poland; esterabakinowska@gmail.com (E.B.); wojciech.biniek6@gmail.com (W.J.B.); kajetan.kielbowski@onet.pl (K.K.); konradszewczyk27@gmail.com (K.S.); hannaostalowska@gmail.com (H.O.); leciejzuzanna@gmail.com (Z.L.); 2 Doctoral School, Poznan University of Medical Sciences, 60-812 Poznan, Poland; kamil.dyrka@ump.edu.pl; 3 Department of Pediatric Endocrinology and Rheumatology, Institute of Pediatrics, University of Medical Sciences, 60-572 Poznan, Poland

**Keywords:** diabetes mellitus, stem cells, mesenchymal stem cells, pancreatic differentiation, induced pluripotent stem cells

## Abstract

Diabetes mellitus is a chronic and progressive metabolic disorder associated with abnormal blood glucose levels. The term involves several diseases with different pathophysiology mechanisms and treatment strategies. Stem cell-based treatments represent an emerging strategy for patients with diabetes mellitus with severe pancreatic insufficiency and poor glycemic control. Over the last 20 years, researchers have investigated mesenchymal stem cell infusion and the transplantation of stem cell-derived β cells and islet tissues. This review aims to comprehensively discuss the latest advances in the field of stem cell use in diabetes, including clinical studies and preclinical experiments aiming at improving the efficacy and safety of stem cell use.

## 1. Introduction

Diabetes mellitus (DM) is a term used to describe a group of metabolic diseases characterized by hyperglycemia. If left untreated, it can result in organ-related complications, particularly nephropathy, retinopathy, and neuropathy. Type 1 DM (T1DM) is associated with insulin deficiency due to autoimmune pancreatic β-cell destruction, whereas T2DM manifests as progressive dysfunction of β-cells, leading to decreased insulin secretion and insulin resistance [1,2], often coexisting with obesity [3]. Diabetes may also be first recognized or have its onset during pregnancy, presenting as abnormal glucose tolerance [4].

The purpose of treatment is not only to rebalance glucose levels, but also to normalize blood pressure, body mass, and lipid profile. Patient education, alongside lifestyle modification, may reduce the risk of disease complications and contribute beneficially to the treatment process [5]. Destruction of the pancreatic islets in T1DM requires lifelong insulin administration via multiple daily subcutaneous injections or continuous delivery systems [6]. Glucose-lowering medications constitute the baseline treatment for T2DM [7]. These agents are widely known by scientists and clinicians; they can sensitize peripheral tissues to insulin, increase glucose secretion with urine, and induce a central effect to regulate satiety, among others. Despite the relatively large arsenal for managing DM, current methods of treatment face several challenges. For insulin treatment, there is a psychological aversion to starting injections. Furthermore, poor adherence to insulin therapy can cause diabetic ketoacidosis or hypoglycemic episodes, both of which can be life-threatening [6,8]. Agents used in the treatment of T2DM represent a relatively safe group, with examples of adverse events including gastrointestinal events in the case of glucagon-like peptide-1 (GLP-1) analogs or urinary tract infections with sodium-glucose co-transporter 2 (SGLT-2) inhibitors [9]. Aside from adverse events, the treatment of T2DM patients is frequently ineffective because of suboptimal adherence [10]. Moreover, current therapies do not aim at improving pancreatic islet functionality.

Thus, new therapeutic approaches should not only involve improving treatment strategies or advancing current classes of drugs, but also investigating novel treatment methods whose effects would be long-lasting and associated with proper adherence and safety. One of the emerging strategies to treat DM is the use of stem cells. A major advantage of stem cell therapy is the possibility of restoring pancreatic production of insulin, which cannot be achieved using currently approved agents. Following decades of research into potential stem cell use in DM, the present review aims to comprehensively summarize the latest findings and present novel concepts surrounding the cellular treatment of DM.

## 2. Who Can Benefit from Stem Cell Treatment?

Novel ideas, theoretical discussions, and preclinical studies drive our understanding of physiology and pathophysiology forward. They offer environments with flexible boundaries that allow us to test and validate new and exciting approaches. Nevertheless, when we enter the field of clinical medicine, everything changes into sterile, guidelines-based behavior. Any modifications to current recommendations need to be systematically and ethically acceptable, confirmed to be reproducible. One of the first questions we need to ask ourselves is who would be the recipient of new treatment agents? As opposed to cellular or animal disease models, patients are treated with the most optimal therapeutics available. In the case of diabetes, which patients would benefit from stem cell therapy needs to be evaluated. The answer to this question depends on the type of DM being considered. In T1DM, documented hypoglycemia unawareness could be the reason for considering treatment options other than insulin [11]. While modern clinical trials evaluate pharmacological interventions as well [12], the use of stem cells could change the paradigm of T1DM treatment. The pathogenesis of T2DM is different from T1DM and mainly involves insulin resistance. The long-term presence of such resistance eventually leads to loss of endogenous insulin release. The administration of stem cells could prevent or restore endocrine function of the pancreas. To further increase the efficacy of stem cells, researchers are trying to identify predictive biomarkers to increase stem cells to adequate populations. The biomarkers will be explored in further sections of this review.

## 3. Stem Cells in Diabetes Mellitus

### 3.1. Stem Cells

**Figure 1:**
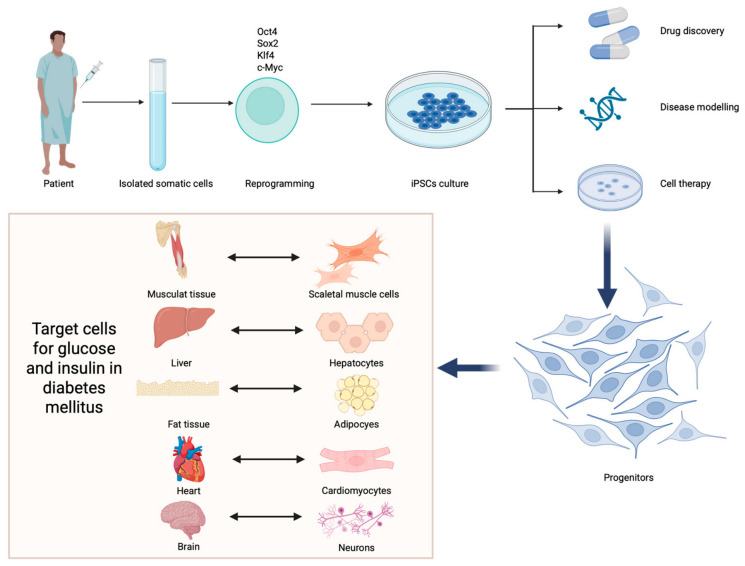
Induced pluripotent stem cells are generated from reprogrammed somatic cells that can be obtained from adult patients. These cells serve as models for drug discovery and disease modeling, and as a cellular therapy. Created in BioRender. Physiology, D. (2026) https://BioRender.com/domuy2c (accessed on 10 May 2026).

“Stem cells” is a broad term that describes several subtypes of cells with different backgrounds and capabilities. Pluripotent stem cells, like embryonic stem cells (ESCs), are characterized as having the possibility of differentiating into various cell lineages with high self-regenerating potential. The drawback of ESCs is their limited collection potential, which is accompanied by ethical issues. Induced pluripotent stem cells (iPSCs) are somatic cells that were reprogrammed to become pluripotent. This mechanism can bypass the abovementioned issue with ESCs, as somatic cells can be obtained from adults (Figure 1). Furthermore, hematopoietic and non-hematopoietic stem cells can also be collected from adults, and their regenerative capabilities have been widely studied. These cells can be collected from the bone marrow, peripheral blood, the liver, adipose tissue or the skin. Furthermore, mesenchymal stem cells (MSCs) can also be obtained from amniotic fluid or the placenta [13]. Stem cells can be collected from and administered to the same person (autologous transplantation) or the procedure can involve collecting cells from other donors (allogeneic transplantation).

### 3.2. Stem Cells in T1DM

T1DM is characterized by the progressive autoimmune destruction of pancreatic insulin-producing beta (β) cells, driven primarily by autoreactive CD4^+^ and CD8^+^ T lymphocytes, B cells, macrophages, and dendritic cells (DCs) that recognize the islet autoantigens presented via MHC class I and II molecules. Stem cell-based therapies address this pathology through two complementary and partially overlapping mechanisms: direct beta-cell regeneration via differentiation of PSCs, and immune modulation predominantly mediated by MSCs and hematopoietic stem cells (HSCs) [14]. Thus, pancreatic transplantation was originally thought of as a rescue procedure for patients with T1DM. While successful transplantation is beneficial for patients, there are several risks and limitations to this procedure. For instance, it requires appropriate recipient selection, and surgery should be performed by experienced team in a center that carries out an adequate volume of such procedures [15]. Moreover, graft survival should be considered. Depending on the report, 5-year survival ranges from 50% to 80% [16,17]. Cellular therapies could offer a different perspective on the treatment of diabetes (Figure 1). It is worth noting that it is now considered that this procedure should be available for patients with both types of diabetes [18].

The ability to use stem cells to restore insulin production was first considered decades ago. For instance, in the year 2000, Soria et al. [19] demonstrated that the tansplantation of insulin-secreting cell clusters obtained from ESCs could improve glycemic control in streptozotocin-induced diabetic mice. Since then, numerous investigators have continued to examine the potential of stem cells in treating diabetes. An interesting method was proposed by Yi et al. [20]. Researchers utilized biliary tree stem cells to form graft patches that were transplanted on the surface of the pancreas in T1DM mice models. The results demonstrated that the patches lowered blood glucose levels and increased the production of insulin.

In 2023, an allogeneic islet product known as donislecel was approved by the FDA for the treatment of T1DM. The studied population involved patients that were unable to reach the target levels of glycated hemoglobin. The agent was made individually from a deceased donor to a single recipient. During clinical studies, participants could receive up to three infusions, provided insulin independence was not achieved. In the clinical evaluation of 30 patients, 21 reached insulin independence for at least 12 months. Eleven patients maintained independence for a period of 1 to 5 years. Importantly, the use of donislecel is associated with the occurrence of adverse events that result from the product itself (immunogenicity), the type of administration (infusion into the portal vein), and long-term immunosuppression [21]. Recent studies focused on evaluating the potential of stem cell-derived islets. For instance, Wang et al. [22] described the clinical use of chemically induced PSC-derived islets in 25-year-old T1DM patients with a history of two liver transplantations and a complete pancreatic transplantation. The pancreatic graft needed to be removed due to thrombotic complications one year after the procedure. Generated islets were introduced underneath the abdominal anterior rectus sheath. At 75 days after the procedure, the patient achieved insulin independence and a major increase in C-peptide concentrations. Importantly, the authors did not report any severe treatment-related adverse events [22]. The promising results described in this paper highlight highly advanced and novel techniques that could be reserved for the most difficult cases. In this young patient, all prior conventional methods failed and the successful use of differentiated islets led to a major improvement in life quality. Nevertheless, several issues must be acknowledged when using similar approaches. Firstly, long-term safety and efficacy of such methods is currently unknown. Secondly, researchers in the above-mentioned study performed transplantation experiments with differentiated islets in 244 mice to ensure the safety and efficacy of this clinical approach. The idea of performing animal experiments prior to clinical use could be used in future protocols to reduce safety concerns.

In other studies, the efficacy of pancreatic endoderm cells (PECs) obtained through the differentiation of ESCs was evaluated. Initially, the cells were implemented in an immunoprotective device, but the immune response to the device was thought to cause inconsistent cell survival [23]. To increase cell survival and evade immune response, PECs were manufactured within permeable devices while the participants were required to receive immunosuppression. Ramzy et al. described the results of a clinical study in which these devices were implanted in the forearm and abdominal wall of patients with T1DM. The authors report reduced insulin dose requirements, significantly more time spent within the glycemic target range, and increased hypoglycemia awareness [11]. Keymeulen et al. [24] also reported the use of PECs encapsulated within permeable devices. Three out of ten patients achieved a predefined increase in C-peptide secretion after 6 months of implantation. Thus, donislecel demonstrated the efficacy of islet products obtained from deceased donors while PECs focused on evading immune response to increase the survival of transplanted cells.

In 2025, a major clinical trial (FORWARD) addressed the efficacy of allogeneic stem cell-derived differentiated islets known as zimislecel in patients with T1DM. The cells were introduced in a single infusion, which was supplemented with immunosuppressive treatment. At the follow-up, researchers observed a gradual improvement in endogenous insulin levels. Furthermore, after the cell infusion, glycemic control considerably improved, as all participants spent more than 70% of the time in the target range of glucose levels, which was in direct contrast to the baseline value (approximately 50%). Strikingly, the treatment resulted in a decrease in insulin dose by 92% between the beginning and after 365 days. Additionally, 10 participants achieved insulin independency [25].

### 3.3. Stem Cells in T2DM

As previously mentioned, it is now considered that stem cell therapy should also be available for patients with T2DM. However, as this has a different pathophysiology compared to T1DM, the adequate point at which to administer stem cells in this population needs to be evaluated. T2DM is associated with chronic insulin resistance and gradual loss of insulin secretion by the pancreas. Whether stem cell therapy is used for patients with preserved pancreatic function or with total pancreatic insufficiency should be considered. At the current level of knowledge in this area, we are beginning to realize that residual function of the pancreas could be a positive prognostic factor in stem cell treatment. In 2023, Wang et al. published their experience with using umbilical cord MSCs in T2DM patients. Their main inclusion criteria involved patients diagnosed for <20 years, with HbA1c levels between 7.0% and 12.0%, with inadequate control by insulin therapy, and with fasting C-peptide concentrations of ≥1 ng/mL. Firstly, the authors demonstrated that target HbA1c levels were obtained by 59.5% of patients at 9 weeks. Secondly, they observed that patients with higher levels of C-peptide and lower baseline HbA1c had a better response to the stem cell therapy [26,27]. These results suggest that a functioning pancreas has a positive effect on stem cells. Perhaps, the presence of insulin-secreting cells creates an adequate environment for stem cells to differentiate towards pancreatic beta cells. Furthermore, residual pancreatic function could alter the immunoregulatory profile of MSCs. Lian et al. [28] published the results of a phase 2 clinical trial that studied the safety of human umbilical cord MSCs in T2DM patients. Approximately 17% of the participants in the study group experienced transient fever and fatigue. Nocturnal hypoglycemia was noted in one patient. At different timepoints, researchers also observed reduced levels of lymphocytes and higher concentrations of platelets in the participants from this study group. In addition, alterations in the coagulation system were noted (higher levels of D-dimers and fibrinogen) [28]. While cell-based therapies did not demonstrate major safety concerns, the study warrants close monitoring regarding coagulation functionality. Perhaps, such patients require anti-thrombotic prophylaxis within the periods in which coagulation abnormalities are observed. The issue of potential thrombosis during treatment will be better studied in clinical trials with large samples.

A double cell-based approach has also been studied. This strategy merges the functionality of stem cells with the benefits of other cellular populations. For instance, combining isolated islets with adipose-derived MSCs promoted cell engraftment and a reduction in hyperglycemia in animal models [29]. In an analysis by Wu et al. [30], researchers utilized both MSCs and mononuclear cells in T2DM patients. Their report offers significant scientific merits as it described 8 years of follow-up. At the final timepoint, cellular therapies demonstrated a significantly higher C-peptide area under curve compared to the control group. Furthermore, double cell therapy was associated with a 19.2% increment compared to 5.9% in the group that received mononuclear cells only. Regarding HbA1c, cellular methods significantly decreased levels at 1 year of follow-up. At 8 years, a return of high glycemic hemoglobin values was observed, but the results were still lower than in the control group. Beneficial effects were also observed regarding fasting blood glucose and insulin dosage requirement 1 year after treatment. At 8 years, the values returned to baseline but were still more satisfactory than those in the control group [30]. Perhaps, the repeated application of cells would result in more pronounced and long-lasting effects. Nevertheless, the recurrent administration of stem cells requires careful and detailed safety monitoring. Another method that was investigated included a combination of bone-marrow-derived mesenchymal stem cells with endothelial cell progenitors. In the study by Guzman and colleagues [31], researchers performed six infusions of this cellular combination in five patients with T2DM. A remarkable response in terms of fasting blood glucose, glycated hemoglobin, blood urea nitrogen and creatinine was observed in one patient. Future investigations into predictive biomarkers should be able to properly identify the individuals most likely to benefit from cellular therapies. While stem cell-differentiated islets represent a method that is more commonly studied in patients with T1DM, Wu and colleagues [32] published a report of the use of this method in a patient with T2DM with impaired insulin secretion. Specifically, the authors performed an intrahepatic administration of islets generated in vitro from autologous endodermal stem cells. Prior to clinical use, their efficacy and safety were evaluated in streptozotocin-induced diabetic mouse and monkey models. After the procedure, time in the tight target range steadily increased until it reached 99% after week 32. Furthermore, insulin independence was achieved as early as week 11 and no episodes of severe hyperglycemia or hypoglycemia occurred for 116 weeks. Despite the significant optimism of the paper, critical concerns were also raised. These included issues regarding proper T2DM diagnosis, glycemic control interpretation, and the contribution of the transplantation to the final clinical outcomes [33].

**Figure 2:**
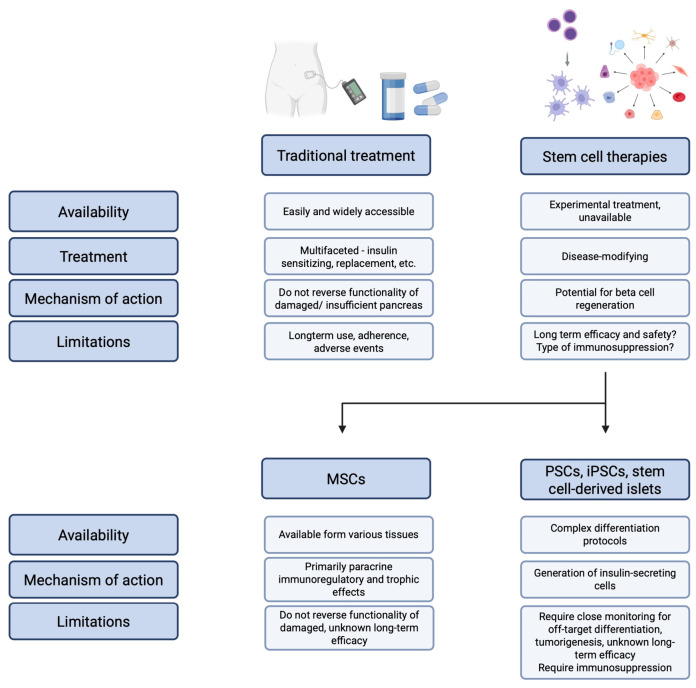
General comparison of standard anti-diabetic medications with stem cell-based approaches. Created in BioRender. Ostalowska, H. (2026) https://BioRender.com/8o4mqvm (accessed on 10 May 2026).

In 2025, Kashbour and colleagues [34] performed a meta-analysis of randomized clinical trials that studied the role of MSCs in the treatment of T1DM and T2DM. The authors included a total of 13 studies, with over 500 patients. The researchers analyzed several parameters, including HbA1c, fasting and postprandial blood glucose, fasting and stimulated C-peptide, insulin requirement, and HOMA scores at 3, 6, and 12 months post treatment. At the third timepoint, the use of stem cell therapy was associated with significantly reduced HbA1c, postprandial blood glucose, increased fasting C-peptide levels, and lower insulin requirement [34]. Therefore, the pooled summary of included trials confirms the potential short-term clinical efficacy of using stem cells. Another important aspect is the safety of such procedures. An overall comparison of stem cells to the classic agents used in the treatment of DM is presented in Figure 2.

### 3.4. How Does Diabetes Affect Stem Cells?

In cases of autologous stem cell administration, potential cellular alterations induced by the disease should be further examined. Intuitively, the cells of individuals with chronic, progressive, and inflammatory diseases can show abnormalities. Recently, an abstract by Kim et al. [35] demonstrated that even MSCs in utero can respond to diabetes of the mother, suggesting that metabolic alterations during embryo development can affect stem cells. In an elegant comparison of stem cells obtained from the peripancreatic adipose tissue of patients with T2DM and healthy controls, several differences were spotted. These included abnormal adipogenic and osteogenic differentiation potential, impaired cell cycle progression, and altered levels of several cytokines in cells of diabetics [36]. In another study, high-glucose stimulation of placenta-derived MSCs also suppressed cell cycle progression and proliferation, together with inducing replicative senescence [37]. To the best of our knowledge, the clinical effect of these differences is currently unknown. Furthermore, we do not know if the tissue of origin of collected stem cells will influence their behavior. Perhaps, adipose tissue obtained from the peripancreatic space is more affected by the disease. However, the periodontal ligament stem cells of T2DM patients also show abnormalities. In a recent report by Chen et al. [38], the authors observed altered autophagy in cells obtained from diabetes patients. Moreover, research on whether the correction of identified abnormalities would translate into improved clinical outcomes is ongoing. However, dapagliflozin, the SGLT2 inhibitor that acts primarily in the kidneys, was also found to promote the expression of MAFA in differentiated β-cells, suggesting that it can promote the functionality of insulin secreting cells [39]. Moreover, the preconditioning of adipose tissue-derived MSCs obtained from T2DM patients with basic fibroblast growth factor could improve their proliferation and migration [40]. Similarly, the conditioning of MSCs obtained from DM patients with a condition medium of healthy MSCs could suppress the inflammatory phenotype of the former cells [41]. These encouraging results suggest that the stem cells obtained for a future autologous treatment from DM patients could be modulated to restore their primary behavior, thus increasing the chances of successful therapy.

## 4. How Could Stem Cell Therapy Be Improved?

Stem cell therapy in patients with DM aims to regenerate insulin-producing cells and modulate immunity to limit inflammatory response present in diabetes. To achieve these goals, we can increase the anti-diabetic efficacy of stem cells themselves, enhance their engraftment and survival, suppress autoimmunity, and monitor and improve their safety profile.

To begin with, cell preconditioning can modulate stem cells’ functionality. Such an intervention is usually tested ex vivo. Nevertheless, if autologous stem cell therapy is considered, any pharmacological intervention prior to stem cell therapy qualification could affect post-transplant effects. In an interesting study by Park et al. [42], the authors noticed that patients with T2DM and coronary artery disease treated with GLP-1 analogs have higher levels of circulating vascular progenitor cells. Furthermore, a 6-month semaglutide treatment was found to increase the proliferation and adipogenesis of adipose-derived stem cells [43]. Perhaps, similar observations could be made regarding MSCs or iPSCs obtained from patients treated with these agents. Ex vivo preconditioning was also proven to increase the antidiabetic efficacy of stem cells. For example, adipose tissue-derived MSCs conditioned in a diabetic environment was associated with greater antidiabetic activity in animal experiments [44]. Interestingly, preconditioning with metformin also resulted in increased insulin levels and decreased glucose concentration [45].

Immunological rejection of the graft remains the principal barrier to the effective implementation of stem cell-based transplantation therapies for diabetes. This process arises from the expression of the HLA molecules on the surface of transplanted cells, which are rapidly recognized as foreign by the recipient’s immune system [46]. Cytotoxic CD8^+^ T lymphocytes recognize non-self HLA class I molecules, leading to target cell death through the release of perforins and granzymes. In parallel, CD4^+^ T lymphocytes amplify the inflammatory response upon recognition of HLA-derived peptides presented by antigen-presenting cells (APCs), resulting in the increased secretion of pro-inflammatory cytokines. This cascade promotes the heightened recruitment and infiltration of immune cells, thereby accelerating the destruction of transplanted cells. Given the complexity of the mechanisms underlying graft rejection, intensive research efforts are focused on developing strategies that enable durable and functional stem cell transplantation. Among the most promising approaches are a reduction in cellular immunogenicity through genetic modifications, the encapsulation of cells derived from iPSCs, and the creation of three-dimensional microenvironments that mimic the architecture and function of the endocrine pancreas. Such strategies are critical for the long-term survival and proper function of transplanted cells [47].

**Figure 3:**
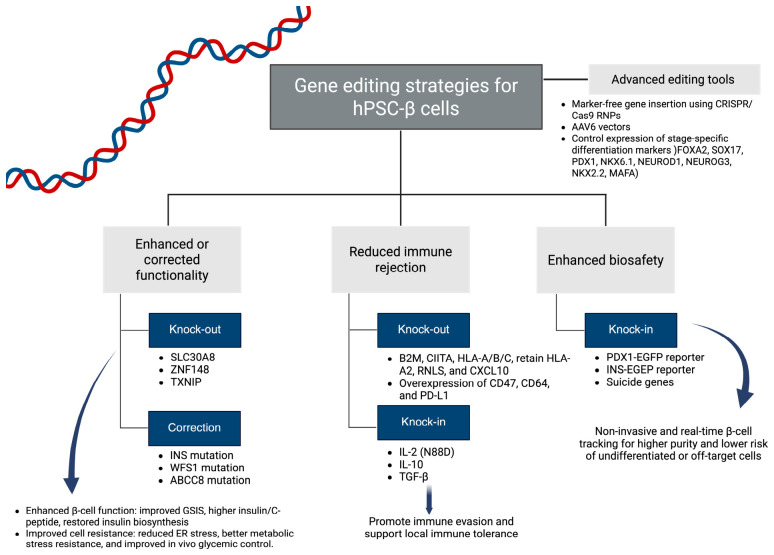
Hypothetical gene editing strategies for stem cell-derived β-cells. Created in BioRender. Physiology, D. (2026) https://BioRender.com/lt338wh (accessed on 10 May 2026).

One of the key directions in the development of therapies based on β cells differentiated from PSCs involves genetic modifications aimed at reducing their immunogenicity prior to transplantation [48]. These strategies focus on interfering with the fundamental mechanisms of immune recognition, enabling transplanted cells to evade immune-mediated elimination without the need for long-term systemic immunosuppression. Recent progress in gene-editing technologies, especially using the CRISPR/Cas9 system, provides highly precise tools to modify or repair PSCs-derived β cells, enabling improved glucose-stimulated insulin secretion (GSIS), reduced immune rejection, and overall enhanced biosafety [49]. CRISPR-mediated gene editing enables modifications such as β2-microglobulin knockout to eliminate MHC class I expression, thereby limiting the activation of cytotoxic CD8^+^ T lymphocytes. Knockout of the class II transactivator suppresses HLA class II expression and reduces CD4^+^ T-cell activation [22,48]. Although these modifications significantly decrease the immunogenicity of PSC-derived cells, they may trigger natural killer (NK) cell responses through a “missing-self” mechanism. This limitation has prompted the concurrent introduction of inhibitory ligands such as HLA-E, HLA-G, and CD47 [22,48]. The presence of these ligands delivers inhibitory signals to NK cells and macrophages, further reducing the risk of graft elimination. In preclinical studies, multigene modification strategies of this type resulted in the long-term survival of hPSC-derived grafts without evidence of active immune responses. PD-L1 and HLA-E overexpression collectively promote immune evasion by suppressing cytotoxic T-cell and NK-cell activity. In this context, modulation of the chemokine CXCL10, which is responsible for recruiting T lymphocytes and NK cells, has been shown to reduce inflammatory infiltration and prolong graft survival in in vivo models [22]. The CRISPR/Cas9-mediated correction of pathogenic insulin (*INS*) or wolfram syndrome 1 (*WFS1*) mutations in patient-derived iPSCs markedly improved insulin biosynthesis and secretion in the resulting iPSC-β cells [46]. In another example, CRISPR/Cas9 knockout of *ZNF148* in ESCs produced ESC-derived β-cells with markedly improved insulin secretion, showing a nearly threefold increase in C-peptide release under high-glucose conditions [50]. Moreover, a homozygous start codon mutation in patient-derived iPSCs was corrected with CRISPR/Cas9 and a single-stranded oligonucleotide donor by exploiting the homology-directed repair pathway [51]. Collectively, immune-evasive modifications aim to transform hPSC-derived cells into therapeutic products with reduced immunogenicity, capable of evading both adaptive and innate immune recognition. The integration of HLA modulation, NK cell-inhibitory signaling, and immune checkpoint regulation currently forms the foundation for the design of hypoimmunogenic cellular platforms (Figure 3).

Apart from genetic modifications, epigenetic reprogramming can also be used to modulate cell plasticity and differentiation potential. Epigenetics involves modification of gene expression without altering the DNA sequence. Classic mechanisms of epigenetic regulation include DNA methylation, histone modifications, or the activity of non-coding RNA. The use of epigenetic factors or modulators can induce epigenetic cell conversion. Researchers described the use of 5-azacytidine (5-AZA), a DNA de-methylator, to change cell plasticity and lineages. For instance, a combination of platelet-derived growth factor–AB and 5-AZA has been demonstrated to induce multipotency in osteocytes and adipocytes [52]. In the case of insulin secreting cells, Brevini et al. [53] utilized 5-AZA in the skin fibroblasts. This process enhanced cell plasticity, allowed these cells to be differentiated into pancreatic cells. Interestingly, the use of 5-AZA was also shown to increase the efficacy of β-cell differentiation using synthetic mRNA molecules of β-cell factors [54].

Transplanted β cells are exposed to complex host immune responses. Graft rejection results from the coordinated action of innate and adaptive immune mechanisms, including the activation of T lymphocytes, NK cells, and macrophages, as well as the presence of a pro-inflammatory cytokine milieu characterized by interferon gamma and TNF-α [46,55]. A critical component of this response is the early inflammatory phase, which promotes immune cell recruitment to the transplantation site and may lead to the rapid loss of a substantial proportion of transplanted cells [55]. Even when graft immunogenicity is reduced, a pro-inflammatory local microenvironment can sustain immune activation and contribute to progressive graft dysfunction. One approach aimed at modulating this response is local immunomodulation at the transplantation site. Presentation of the Fas ligand (FasL) on the surface of transplanted islets or biomaterials induces apoptosis of infiltrating Fas-expressing T lymphocytes, thereby limiting effector immune responses and promoting the establishment of localized immune privilege [56]. In animal models, this strategy enabled long-term graft survival without the need for chronic systemic immunosuppression. Complementary immunomodulatory strategies include tissue engineering approaches, particularly the encapsulation of hPSC-derived cells within semipermeable biomaterials. Encapsulation limits direct contact between the graft and immune cells; however, it does not fully eliminate the effects of pro-inflammatory cytokines or challenges related to foreign body responses and compromised microenvironmental conditions, which may restrict the long-term efficacy of this approach [48,55]. From a clinical perspective, post-transplant immunomodulatory strategies focus on limiting local inflammation and preserving graft function during the critical early period following implantation. Despite promising preclinical outcomes, achieving full control of host immune responses without systemic immunosuppression remains a significant challenge. Genetic editing represents another mechanism that could regulate autoimmunity. The presence of regulatory T cells can promote immunosuppression, which can be beneficial in the context of transplantation [57]. Interestingly, Filatov and colleagues [58] showed that ESC-derived islets engineered to secrete CCL22 could promote the infiltration of Tregs towards transplanted cells in vivo. Similar mechanisms could be utilized to increase the survival of grafts. In an abstract presented by Zhao et al. [59], the authors promoted immune evasion by genetically modifying ESCs to express immunomodulatory molecules, such as PD-L1. Interestingly, culturing these cells with immune cells could inhibit their pro-inflammatory activation. Perhaps, such strategies could influence immunosuppressive regimens administered in recipients of cellular-based treatments.

Among the different stem cell types, ESCs and iPSCs exhibit the highest risk of tumorigenicity, with comparable levels of risk reported for both populations [48]. The lowest risk of malignant transformation is observed with MSCs, reflecting their limited differentiation potential and lack of pluripotency. The primary concern associated with pluripotent stem cell-based therapies is the risk of teratoma formation, which arises from the presence of residual undifferentiated pluripotent stem cells within the transplanted cell product [60]. These cells express key pluripotency factors such as OCT4, SOX2, and NANOG and may retain their capacity for uncontrolled proliferation if not completely eliminated during differentiation and cell manufacturing processes [46]. Although, to the best of our knowledge, no clinical reports have confirmed teratoma formation in stem cell-based therapies for T1DM to date, the development of robust safety strategies remains a critical aspect of therapy design. Recently, in an interesting study by Wu et al. [61], the authors analyzed undifferentiated cells in islet cells derived from chemically induced PSCs. Researchers utilized long non-coding RNA to evaluate residual undifferentiated cells present in the islet cells. This and similar approaches could be implemented to monitor the safety of stem cell-based therapies. One promising approach involves the generation of hypoimmunogenic cell lines equipped with so-called safety switches, enabling the selective elimination of transplanted cells in the event of adverse effects [62]. These systems rely on the induction of suicide genes, such as herpes simplex virus thymidine kinase, which converts the prodrug ganciclovir into a toxic nucleotide analog, resulting in DNA synthesis termination and selective cell death. An alternative strategy is the iCasp9 system, in which the activation of pro-apoptotic caspase-9 occurs following the administration of the small-molecule ligand AP1903 (rimiducid), enabling the rapid and controlled induction of apoptosis in transplanted cells. In a study by Wang et al., tumorigenicity risk was monitored through assessment of tumor markers and regular imaging studies. During one year of follow-up, no evidence of teratoma formation or other graft-related malignancies was observed [22]. As the use of stem cells in DM is still an emerging field, there are currently no clinical guidelines on the duration of oncological monitoring. In preclinical settings of various disease models and indications, the observation period is stated to range from 10 to 40 weeks [63]. Importantly, the specific time depends on the disease context and the type of cellular product. However, in clinical settings such monitoring would need to be much more extended. In addition, it should be emphasized that one of the most serious adverse events reported in clinical studies involving stem cell therapies was severe neutropenia, leading to increased susceptibility to opportunistic infections. This represents a significant safety limitation and underscores the need for the further optimization of therapeutic protocols [25].

The most significant limitation of stem cell-based therapies remains the requirement for long-term, often lifelong, immunosuppression to prevent graft rejection [55]. Such treatment is associated with substantial systemic toxicity and an increased risk of infections and malignancies, markedly limiting the safety profile and broad clinical applicability of these therapies. Although many of the strategies described effectively attenuate immune responses associated with islet transplantation and define directions for future therapeutic development, immunosuppression currently remains essential to ensure graft survival and proper function. The goal of immunosuppressive regimens is to achieve durable immune protection using the lowest possible drug doses in order to minimize adverse effects. In addition, given the critical role of early inflammatory responses in graft loss, the use of anti-inflammatory agents may reduce damage induced by pro-inflammatory factors and improve islet function during the initial post-transplantation period [64]. Additionally, implanted cells can undergo a post-transplantation apoptosis. To limit this mechanism, an interesting mechanism was suggested by Baker et al. [65]. In in vitro experiments, researchers showed that the delivery of BCL-2 can increase the viability of β-cells, thus increasing the chances of a successful procedure.

## 5. Stem Cells and Diabetic Complications

Cardiovascular disease is the leading cause of death in patients with DM [66]. Chronic hyperglycemia disrupts cardiac metabolism through multiple mechanisms, including oxidative stress, persistent inflammation, insulin resistance, the accumulation of advanced glycation end products (AGEs), and overactivation of the hexosamine biosynthetic pathway. Myocardial contractile dysfunction most commonly arises from atherosclerosis and diabetic cardiomyopathy (DCM), which can ultimately lead to sudden cardiac death [67]. iPSCs can provide a renewable source of vascular cells for investigating the mechanisms underlying hyperglycemia and insulin resistance, as well as for developing personalized therapies to prevent cardiovascular disease in diabetic patients [68]. Studies have shown that exposing iPSCs-derived cardiomyocytes to sustained insulin in the absence of glucose in vitro can mimic key physiological features of DCM [69]. Gheibi et al. created insulin- and glucose-responsive cells capable of modeling diabetic conditions in vitro, establishing an effective platform for drug testing and personalized therapy [70]. iPSCs-derived cardiomyocytes serve as valuable disease modeling tools for cardiac conditions, including DCM and heart failure, enabling a better understanding of disease pathogenesis and the development of targeted therapies for T2D-related cardiac dysfunction [71]. IR in T2DM affects not only classical insulin target tissues—such as the liver, skeletal muscle, and adipose tissue—but also the brain, cardiomyocytes, and nephrons. In the brain, IR is associated with cognitive decline and neurodegenerative diseases. In cardiomyocytes, it can result in lipotoxicity and impaired cardiac function, while in nephrons, it exacerbates glucose intolerance [72]. Implantation of these cells-engineered to secrete insulin-sensitizing factors, may help restore cardiac function and improve clinical outcomes in patients with IR and DM [73]. Given that IR underlies the development of T2DM, iPSCs offer a promising tool for investigating IR and its associated conditions.

Diabetic neuropathy (DN) is a common and debilitating complication of DM, significantly affecting patient quality of life and contributing to morbidity and mortality [74]. iPSCs-derived neurons from diabetic patients have been used to model DN, revealing disruptions in calcium homeostasis, oxidative stress, and the activation of neuroinflammatory pathways. Preclinical studies have shown potential to enhance nerve function and alleviate peripheral neuropathy symptoms [75]. In addition, PSCs-derived Schwann cells (also originating from iPSCs) are highly sensitive to glucose-induced glucotoxicity, providing a valuable platform for investigating the mechanisms underlying diabetic peripheral neuropathy [76]. Additionally, recent studies show that iPSCs-derived Müller glial cells can treat retinal damage, improving vision in glaucoma and retinitis pigmentosa [77].

Diabetic foot ulcers (DFUs) are complications of DM, representing a major cause of morbidity, lower-limb amputation, and healthcare burden worldwide. DFUs affects approximately 6.3% of patients; the number of affected patients is lower in DM1 than DM2 [78]. Chronic hyperglycemia can cause a disruption in vascular integrity, which compromises wound healing and reduces immune function. Traditional therapeutic methods, such as wound debridement, vascular reconstruction, and infection control, often fail to achieve satisfactory outcomes, emphasizing the necessity of advanced regenerative interventions. Surgical interventions are limited by technical difficulties, including recurrent vascular occasion and unsuccessful recanalization [79]. In recent years, stem cell-based therapies have gained recognition as a promising approach for DFUs. These cells exhibit exceptional self-renewal capacity and the potential to differentiate into multiple lineages, while releasing paracrine mediators that regulate inflammation, stimulate angiogenesis, and support tissue repair. Several stem cell populations, including MSCs, and bone marrow-derived progenitors, have shown encouraging therapeutic outcomes. Studies show potential for wound closure and restore functional tissue.

The meta-analysis by Mei et al. provided clinical insight into the potential of stem cell therapy for patients with DM. MSCs treatment increased rates of completely healed ulcers, with better outcomes in smaller lesions under 5 cm^2^, but demonstrated no significant effect in ulcers greater than 5 cm^2^ [80]. Zeng et al. evaluated therapeutic efficacy of placental-derived MSC hydrogels in the treatment of DFUs. Their findings indicated that MSC–hydrogel application shortened overall healing time, significantly reduced wound size withing three weeks, and promoted the formation of dense granulation tissue conductive to wound repair [81]. Moreover, MSCs suppress the activity of inflammatory cells while promoting regulatory T cells’ presence, which help maintain immune tolerance and homeostasis. Additionally, MSCs increase the population of M2 anti-inflammatory macrophages and decrease M1 macrophages which induce tissue injury and impede angiogenesis [82]. In the meta-analysis wound healing rates improved markedly across all stem cell types, with peripheral blood-derived stem cells exhibiting the greatest therapeutic effect. Adipose-derived stem cells ranked second in efficacy, followed by umbilical-cord-derived stem cells, bone marrow-derived stem cells, and stem cells from other sources. However, only cells obtained from umbilical cord provided statistical significance. The result may point to their stronger regenerative potential and more consistent biological activity. Larger studies are needed to confirm comparative efficacy among stem cell sources [83]. Another meta-analysis demonstrated that no statistically significant differences in wound healing rates was found among MSCs derived from amniotic membrane, adipose tissue, umbilical cod, or bone marrow at evaluation intervals of days 7–8, 10–12, and 12–14 [84].

## 6. Conclusions and Future Perspectives

Cellular-based approaches for patients with diabetes are promising. A considerable number of reports confirm their efficacy, with improvements in glycemia control, insulin levels and exogenous insulin dose requirements. Nevertheless, they have important limitations that need to be addressed in future evaluations. Firstly, current evidence mostly comes from small-sample studies. Larger trials are required to properly evaluate safety. Secondly, long-term efficacy needs to be further evaluated, as only a few studies provide a follow-up period of more than 5 years. Immunosuppression represents another topic of concern. It is widely known that long-term immunosuppression increases the risk of serious infections or metabolic disorders. The decision to introduce cell-based approaches will need to take into consideration potential benefits and risks associated with infusion strategy and immunosuppression therapy. Future investigations into products that would minimize immunosuppression are needed. Importantly, specific protocols and guidelines should be developed for each class of transplanted cells, as their functionality, properties, and potential adverse events differ. These risk-to-effect considerations should be specifically strict in the case of patients with T2DM. Currently, we possess a large arsenal of effective antidiabetic medications that are registered for the treatment of T2DM. The decision to initiate stem cell therapies should only be considered in insulin-dependent patients with severe pancreatic insufficiency and poor glycemic control. However, there could be another use of stem cells in the area of diabetes. In an in vivo experiment by Yarani and colleagues [85], the authors demonstrate that MSCs could be used to prevent the development of T2DM. Perhaps, if studies further expand in this direction, stem cells could be used to inhibit the progression of metabolic alterations leading to diabetes.

## Data Availability

No new data were created or analyzed in this study. Data sharing is not applicable to this article.

## Abbreviations

DM	Diabetes mellitus
T1DM	Type 1 diabetes mellitus
T2DM	Type 2 diabetes mellitus
TZDs	Thiazolidinediones
DPP-4i	Dipeptidyl peptidase-4 inhibitor
PSCs	Pluripotent stem cells
iPSCs	Induced pluripotent stem cells
PECs	Pancreatic endodermal cells
PPI	Pancreatic progenitor cells
MODY	Maturity-onset diabetes of the young
GSIS	Glucose-stimulated insulin secretion
Tregs	Regulatory T cells
HUVECs	Human umbilical vein endothelial cells
PBMCs	Peripheral blood mononuclear cells
IR	Insulin resistance
DCM	Diabetic cardiomyopathy
DN	Diabetic neuropathy
DFU	Diabetic foot ulcer
